# Editorial: Oligomers in amyloid-associated diseases—structural properties and toxicity

**DOI:** 10.3389/fmolb.2023.1215340

**Published:** 2023-06-21

**Authors:** Neville Vassallo, Sandrine Ongeri, Alfonso De Simone

**Affiliations:** ^1^ Department of Physiology and Biochemistry, Faculty of Medicine and Surgery, University of Malta, Msida, Malta; ^2^ Centre for Molecular Medicine and Biobanking, University of Malta, Msida, Malta; ^3^ BioCIS, CNRS, Université Paris-Saclay, Saint Aubin, France; ^4^ Department of Pharmacy, University of Naples Federico II, Naples, Italy; ^5^ Department of Life Sciences, Imperial College London, London, United Kingdom

**Keywords:** proteinopathies, neurodegenerative, amyloid, oligomers, structure, toxicity

The so-called “amyloid proteinopathies” represent a constellation of human diseases associated with the pathological formation and deposition of aberrant insoluble protein aggregates. They include neurodegenerative conditions such as Alzheimer’s, Parkinson’s and Huntington’s diseases, amyotrophic lateral sclerosis, and prion diseases; but may also involve organs and tissues outside the central nervous system, for instance the pancreas in type-2 diabetes mellitus. Collectively, amyloid-associated disorders affect around 50 million people each year worldwide and no cure is essentially known to stop the underlying molecular mechanisms of their origin. These conditions therefore generate significant burden on individuals, societies and healthcare budgets, and there is a burgeoning need for the rational development of effective therapeutic strategies.

A fundamental biophysical process underlying the pathogenesis of proteinopathies concerns the conversion and aggregation of an amyloidogenic protein or peptide from an initial, often structurally disordered, monomeric state into a mature, highly-ordered fibrillar state. Thus, the protein explores multiple intermediate conformations as it transitions along an aggregation continuum to mature fibrils. A fascinating Research Topic in the field concerns the characterization of such a bewildering array of protein aggregate species – posing the key question: which of these myriad entities is/are most relevant for toxicity? Over the past decade, a consensus has emerged that small, partially-folded metastable species formed early during the aggregation process, collectively termed “oligomers”, represent the most likely pathogenic form. However, large gaps remain in our knowledge about oligomers, not least because of their heterogeneity and their inherent propensity to rapidly transition into higher order aggregates, which makes their isolation highly challenging. Important pending questions, amongst others, include: do oligomers share common structural and physicochemical features; which property determines pathogenicity; can a correlation be made between toxic effects and a specific type of oligomer; what is the diversity of oligomers in samples isolated from patient-derived tissues and biological fluids; and what is their role in trans-cellular spread of disease.

In this Research Topic, we have published four articles dedicated to amyloid oligomer research, of which three are reviews and one an original research paper. It is pertinent to note that all four articles address relatively cutting-edge topics in the field of aberrant protein oligomers, namely: the complex energy landscape of oligomer formation; the phenomenon of cross-talk among amyloid proteins; and huntingtin aggregation in Huntington’s disease, the latter being arguably amongst the least explored amongst the major brain neurodegenerative diseases.

A new prospective into the oligomer field is provided by Muschol and Hoyer. The authors define amyloid oligomers as small, globular assemblies formed at an early stage along the aggregation pathway, exhibiting inherent metastability. Importantly, liquid-liquid phase separation is excluded as a distinct aggregation mechanism. They then proceed to tackle the intriguing problem of understanding the fundamental biophysical processes behind formation of “on-pathway” and “off-pathway” intermediates. The former constitute oligomers that can further accrue into the fibrillar state, whilst the latter accumulate in a local free energy minimum away from fibrillar nucleation. Although off-pathway oligomers do not go on to form fibrils, they may still be cytotoxic and relevant to disease. The authors critically evaluate a wealth of kinetic data from *in vitro* experiments and rationalize oligomer formation into three possible landscapes: 1) on-pathway nucleated polymerization, 2) on-pathway nucleated conformational conversion, and 3) off-pathway oligomerization. Such efforts at unraveling the mechanisms of amyloid oligomer formation are a prerequisite for a comprehensive understanding of the even more complex mechanisms at play *in vivo.*

Several recent studies are providing accumulating evidence that many amyloid disorders are mixed proteinopathies. Copathologies imply cross-talk among amyloid proteins, with molecular interactions and cross-seeding between aggregates. Thus, in another contribution to the current Research Topic, the author surveys the co-assembly of the 40–42 amino acid amyloid-beta (Aβ) peptide with the 140 amino acid α-synuclein (α-syn) protein, which represent the pathological hallmarks of Alzheimer’s disease (AD) and Parkinson’s disease (PD), respectively (Kim). Indeed, the pathological synergy of Aβ-α-syn interactions appears to stem from enhanced oligomerization. Such interactions can occur both intracellularly, e.g. in mitochondria, or in the extracellular space, as demonstrated *in vitro* and *in vivo*. Undoubtedly, the formation of oligomers from co-assembly of Aβ and α-syn further increases the pathological complexity of the resulting aggregate structures. This realization is likely to have important functional consequences for AD/PD pathogenesis and necessitates multitherapies, targeting simultaneously Aβ, α-syn, and possibly tau.

Of the amyloidogenic proteins involved in brain neurodegeneration, the Huntingtin (Htt) protein related to Huntington’s disease (HD) has historically been less extensively investigated. Poly-glutamine (polyQ) extensions in mutant Htt (mHtt) are inherited in an autosomal dominant fashion and result from CAG nucleotide repeat expansions in the *HTT* gene. An increased number of repeats makes the protein more structurally unstable, lowers the age of disease onset and aggravates the severity of the phenotype. While studies have been conducted on mHtt fibrils, less is known about the oligomerization process. As with other amyloid proteinopathies, soluble mHtt oligomers have been proposed to be the toxic species in HD. In their review, Jarosińska and Rüdiger argue in favour of targeting techniques and strategies for treating HD by removing mHtt oligomers, ideally using a highly selective drug at an early stage. These might involve mRNA targeting approaches, or modulating mHtt protein degradation using autophagy. Misfolding and aggregation of the polyQ tract in Htt is computationally simulated by Khaled et al., in all-atom molecular dynamics (MD) simulations, interestingly comparing non-pathogenic (*n =* 23) and pathogenic (*n =* 48) monomers and dimers of the N-terminal Htt (Htt-ex1). Structural insight is hence provided into aggregation pathways of polyQ peptides whilst considering residues flanking the polyQ sections at the N-terminus and C-terminus. Notably, the authors observed evident formation of short β-sheets in the pathogenic monomer.

In conclusion, this Research Topic focuses on a few areas that are being most actively explored in the field of amyloid oligomers. Without doubt, the amyloid community has to increase its efforts to face the as yet unmet challenge of disease-modifying therapy for amyloid proteinopathies, and tackle these diseases at their root cause.

